# Impact of oversedation prevention in ventilated critically ill patients: a randomized trial—the AWARE study

**DOI:** 10.1186/s13613-018-0425-3

**Published:** 2018-09-21

**Authors:** SRLF Trial Group, Bernard de Jonghe, Jérôme Aboab, Nadia Aissaoui, Djillali Annane, Corinne Audoin, Jean-Luc Baudel, Florence Brouard, Alexandre Cambonie, Isabelle Camilatto, Karim Chergui, Vincent Das, Daniel da Silva, Nicolas Devos, Nicolas Deye, Stephan Ehrmann, Frédérique Ganster, Bruno Giraudeau, David Grimaldi, Emmanuelle Gourdin, Antoine Gros, Olfa Hamzaoui, Frédéric Jacobs, Antoine Kimmoun, Jean-Claude Lacherade, Bernard Lambermont, Pierre-François Laterre, Julie Leger, Stéphane Legriel, Lucas Liaudet, Charles-Edouard Luyt, Philippe Michel, Jean-Paul Mira, Xavier Monnet, Grégoire Muller, Michael Piagnerelli, Gaëtan Plantefeve, Jean Reignier, Jean-Damien Ricard, François Vincent, Jugurtha Aliane, Fabienne Plouvier, Alain Mercat, Mohebbi Amoli Abolfazl, Gaëtan Plantefeve, Cédric Cleophax, Karim Chergui, Guillaume Carteaux, Jérôme Aboab, Jean Reignier, Gilles Troche, Laurent Guerin, Patrick Girardie, Emmanuel Vivier, Romain Hernu, Philippe Obbee, Laurence Donetti, Thierry Jacques, Aurélie Cravoisy-Popovic, Thierry Boulain, Qin Lu, Danielle Reuter, Elie Azoulay, Hervé Clavier, Walter Picard, René Robert, Renaud Chouquer, Christophe Girault, Daniel da Silva, Stéphane Merat, Charlotte Quentin, Jean-François Hicter, Maleka Schenck, Sandie Dauriac, Jean-Luc Desmaretz, Hervé Hyvernat, Alexis Soumer, Annabelle Stoclin, Jean-Philippe Rigaud, Alexandre Duguet, Laetitia Bodet-Contentin, Siu-Ming Au, Sébastien Ena

**Affiliations:** Société de Réanimation de Langue Française (SRLF), 48 Avenue Claude Vellefaux, 75010 Paris, France

**Keywords:** Intensive care units, Mechanical ventilation, Sedation, Mortality, Weaning

## Abstract

**Background:**

Although oversedation has been associated with increased morbidity in ventilated critically ill patients, it is unclear whether prevention of oversedation improves mortality. We aimed to assess 90-day mortality in patients receiving a bundle of interventions to prevent oversedation as compared to usual care.

**Methods:**

In this randomized multicentre trial, all adult patients requiring mechanical ventilation for more than 48 h were included. Two groups were compared: patients managed according to usual sedation practices (control), and patients receiving sedation according to an algorithm which provided a gradual multilevel response to pain, agitation, and ventilator dyssynchrony with no specific target to alter consciousness and no use of sedation scale and promoted the use of alternatives to continuous infusion of midazolam or propofol (intervention).

**Results:**

Inclusions were stopped before reaching the planned enrolment. Between 2012 and 2014, 584 patients were included in the intervention group and 590 in the control group. Baseline characteristics were well balanced between groups. Although the use of midazolam and propofol was significantly lower in the intervention group, 90-day mortality was not significantly lower (39.4 vs. 44.2% in the control group, *p* = 0.09). There were no significant differences in 1-year mortality between the two groups. The time to first spontaneous breathing trial and time to successful extubation were significantly shorter in the intervention group than in the control group. These last results should be interpreted with precaution regarding the several limitations of the trial including the early termination.

**Conclusions:**

This underpowered study of severely ill patients was unable to show that a strategy to prevent oversedation could significantly reduce mortality.

*Trial registration* NCT01617265

**Electronic supplementary material:**

The online version of this article (10.1186/s13613-018-0425-3) contains supplementary material, which is available to authorized users.

## Background

Intravenous hypnotics, often combined with morphinics, are commonly used in mechanically ventilated patients in the intensive care unit (ICU) to control pain, agitation, and ensure synchrony with the ventilator [1]. However, the continuous infusion of midazolam or propofol often results in oversedation [2]. Factors involved in oversedation are multiple, including drug pharmacokinetic and pharmacodynamic properties, inadequate objectives in terms of consciousness, and lack of frequent reassessment of patient condition and hypnotic needs. Oversedation has been associated with prolonged mechanical ventilation and higher rates of nosocomial infections, ICU-acquired weakness, and delirium.

Strategies developed to avoid oversedation have been based either on (1) the use of continuous intravenous hypnotics combined with daily interruption of sedatives every 24 h [3], (2) the continuous titration of hypnotics according to predefined goals of comfort and consciousness, with frequent patient assessments and prescription changes [4–7], or (3) the first-intention use of alternatives to continuous intravenous midazolam or propofol [8–10]. These strategies were associated with a reduction in mechanical ventilation duration. Few observational studies have assessed the impact of an oversedation prevention strategy on mortality [10–13]. In a prospective observational study, Shehabi et al. reported that patients with deep sedation (indicated by at least one measurement of Richmond Agitation Sedation Scale [RASS] − 3 to − 5) during the first 48 h of mechanical ventilation were more likely to die in the ICU than patients with a lighter sedation (RASS − 2 to + 1), independently of age, comorbidities, and severity of acute illness [11]. To the best of our knowledge, this observation has not been confirmed in a randomized trial.

The main objective of the present randomized controlled trial was to determine whether a strategy aiming to prevent oversedation could reduce 90-day mortality in critically ill patients requiring mechanical ventilation compared to usual care.

## Methods

In accordance with French law, the study was approved by the institutional review board of Clermont-Ferrand, France, and by the Ethics Committee of the French Intensive Care Society (SRLF, Société de Réanimation de Langue Française). Informed consent or deferred consent was obtained from each patient or his/her legal surrogate. An independent data safety monitoring board had full access to the unblinded data.

### Participants and settings

The study conducted by the SRLF Trial Group was planned as a parallel two-group individually randomized trial. Patients were eligible if they were aged 18 years or more, were receiving invasive mechanical ventilation for < 12 h, and had an expected invasive mechanical ventilation duration > 48 h after randomization. Patients admitted after cardiac arrest, those with neuromuscular disease, tracheostomy, severe intracranial hypertension, status epilepticus, decision to withdraw care, or considered moribund were not included.

### Oversedation prevention (OSP)

**Fig. 1:**
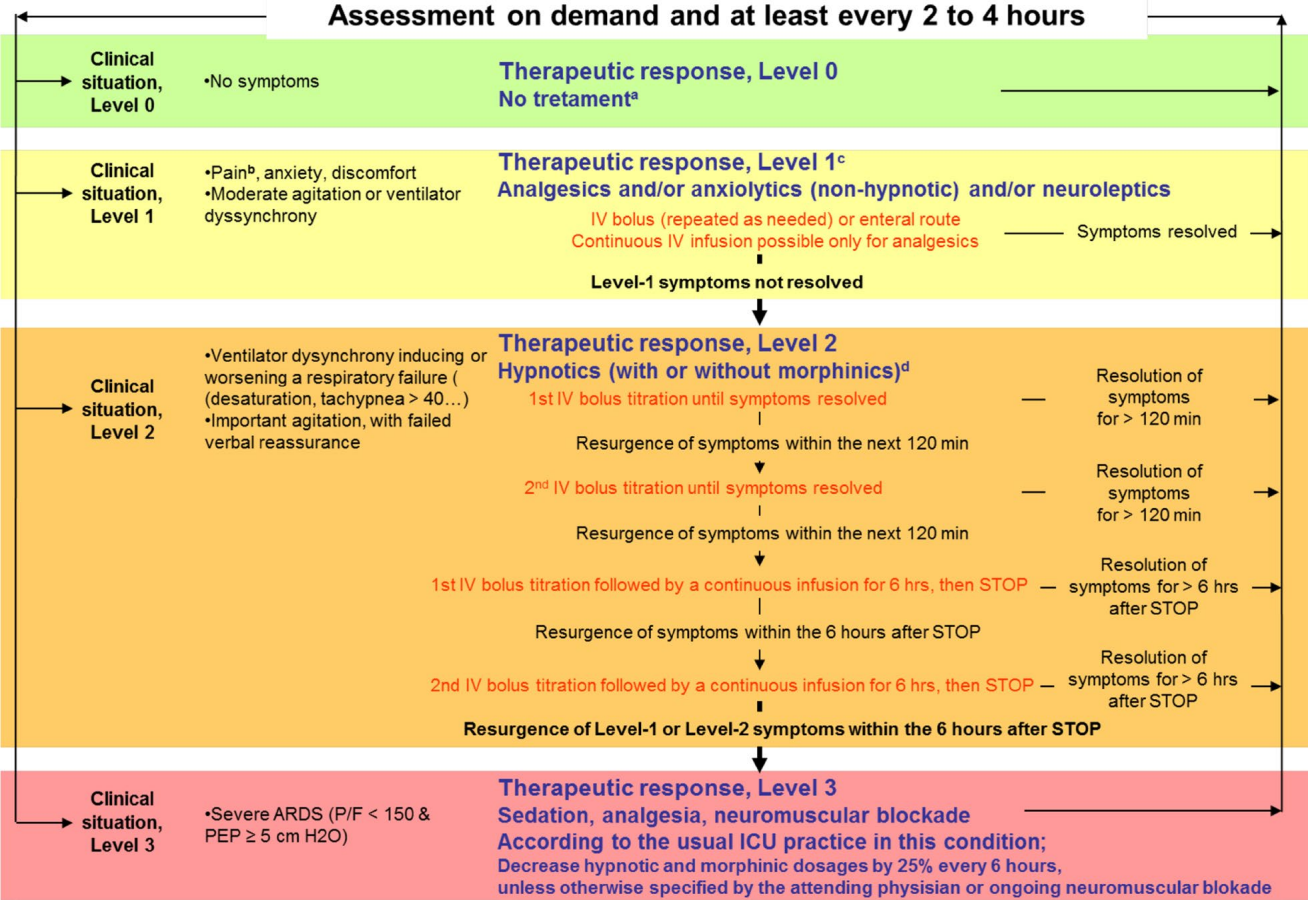
Oversedation prevention (OSP) strategy was centred on patients’ level of agitation, ventilator dyssynchrony, and pain, assessed on a 4-level scale, with gradual on-demand responses, frequent reassessments, and promotion of alternatives to continuous around-the-clock infusion of intravenous hypnotics. These alternatives included frequent (every 6 h) intravenous hypnotic interruptions, intravenous boluses of hypnotics without continuous intravenous infusion, and the use of non-hypnotic drugs, including neuroleptics, hydroxyzine, and anxiolytic benzodiazepines. The choice of the non-hypnotic drugs and their route of administration (intravenous boluses or nasogastric tube) were left to the preference of the attending physician. There was no restriction for the use of morphinics and non-morphinic analgesics. Patient at Level 0, who showed no discomfort, received no treatment, or continuation of a successful level-1 therapeutic response (**a**). Patient at Level 1, with only moderate discomfort, pain, or anticipated procedural pain (**b**), received any form of analgesics as deemed necessary by the attending physician and/or non-hypnotic drugs as well as verbal reassurance and, if appropriate, changing of ventilator settings (**c**). Patients at Level 2, with severe agitation or ventilator dyssynchrony first received repeated intravenous boluses of either propofol or midazolam according to physician preferences, and, if discomfort persisted, 6-h continuous intravenous infusion of midazolam or propofol. This treatment was also applied in case of Level 1 therapy failure (which was maintained or stopped according to physician preference (**d**). Patients at Level 3, with ARDS and a PaO_2_/FiO_2_ ratio < 150 mmHg, were treated with a continuous intravenous infusion of midazolam or propofol, with neuromuscular blocking agents administered according to physician preference. This treatment was also applied in the case of Level 2 therapy failure

The OSP strategy was centred on the identification of patients’ level of agitation, ventilator asynchrony, and pain, on a 4-level scale, with gradual on-demand responses, frequent reassessments, and promotion of alternatives to continuous around-the-clock intravenous hypnotics infusion (Fig. 1). The alternatives to continuous infusion of midazolam or propofol in the intervention group were other benzodiazepines, antipsychotic agents, zolpidem, and hydroxyzine.

There was no aim to alter consciousness, and therefore, no sedation scale was used. However, consciousness alteration could result from treatment of existing agitation, existing ventilator dyssynchrony, or existing or anticipated pain. During two national training and education meetings, the OSP strategy was explained to investigators (usually an ICU doctor and nurse), who in turn implemented the OSP strategy in their own centre. OSP strategy posters were used at the bedside.

### Usual care

Patients in the control group were treated according to the routine sedation practices used in each participating centre, reported in an pre-study survey of sedation practices in France [14]. In both intervention and control groups, the use of dexmedetomidine was not permitted. In both groups, pain was measured according to the current practice in each participating centre. Weaning was conducted according to the French ICU Society guidelines [15].

### Outcomes

The primary study endpoint was 90-day mortality after randomization. Secondary endpoints were day-28, hospital and 1-year mortality, time from randomization to first spontaneous breathing trial, time to successful extubation (defined as absence of invasive mechanical ventilation for 48 consecutive hours). Other outcome criteria are reported in Additional file 1: Appendix 1.

### Randomization, allocation concealment, and blinding

Using a secure, computer-generated, interactive, web-response system available at each study centre, patients were randomly assigned in a 1:1 ratio to study groups. Randomization was stratified by centre using permutation blocks of variable sizes. Sequences were generated by a biostatistician not involved in patient recruitment. Investigators had no access to the randomization list and were not aware of the size of the randomization blocks. Given the very nature of the assessed intervention, blinding of the physicians and nurses was not feasible [16–18]. However, the primary outcome (death at day 90) is an objective one, which counterbalances this lack of blinding [19].

### Sample size

We assumed a mortality rate of 22% in the control group at day 90. To show a 5% absolute reduction in 90-day mortality in the OSP group, with a two-sided type I error of 5% and a power of 90%, the planned enrolment was 2720 patients.

### Statistical analysis

Statistical analyses were conducted in the intention-to-treat population. Data were described using counts and percentages or means and standard deviations (or median and interquartile range). Death proportions were compared using the Chi-square test, and mechanical ventilation-free days were compared using the Wilcoxon test. Time-dependent events were analysed using competing risk models taking into account death and extubation. Description of the occurrence of these events was made using cumulative incidence curves. Cumulative incidence curves were compared between the two groups with the Fine and Gray test; hazard ratios (HRs) and their 95% confidence intervals (95% CIs) were estimated by the competing risk models [20]. For the two patients (one in the OSP group and one in the control group) lost to follow-up for the primary outcome (90-day mortality), imputation of missing data (alive status) was performed. A two-tailed *p* value < 0.05 was considered statistically significant. Statistical analyses were performed using SAS version 9.4 (SAS Institute Inc) and R version 3.2.2 [21].

## Results

### Baseline characteristics

**Fig. 2:**
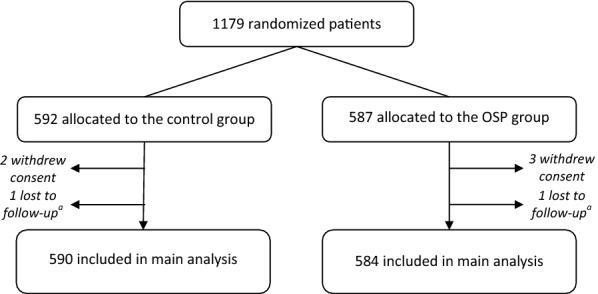
Flowchart. ^a^Patients lost to follow-up: imputation of missing data (alive vital status) was performed. OSP, oversedation prevention

Forty-six ICUs were involved in the study. Of those, 18 (39.1%) were university-affiliated, 15 (32.6%) were medical ICUs, and 31 (67.4%) medical surgical ICUs. Between July 2012 and July 2014, 1179 patients were included and randomized. The nurse/patient ratio was 2.5. The trial was stopped because of a decreasing average per centre recruitment rate, despite considerable help and encouragement. Five patients withdrew their consent and were therefore excluded from analysis, as requested by French law. Two patients (one in each group) were lost for the 90-day follow-up and were arbitrarily considered alive (Fig. 2), leaving 584 patients in the OSP group and 590 in the control group available for the main analysis.

Table 1 shows the patients’ baseline characteristics, which were similar between the two groups. In the OSP and control groups, mean age (standard deviation) was 66 (13) and 67 (14), mean simplified acute physiology score II (SAPS II) was 53.6 (17.8) and 54.4 (18.6), and 54.5 and 53.0% of the patients were receiving norepinephrine at randomization, respectively. Of note, chronic psychotropic medication use did not differ between the two groups, as shown in Table 1. At randomization, more than 65% of the patients were comatose (deep sedation or unarousable), as shown in Table 1. Sedation levels at randomization are reported in Additional file 1: Appendix 2.

**Table 1:** Demographic and baseline characteristics at randomization

	Control (*n* = 590)	Oversedation prevention (*n* = 584)
Age (years), mean (SD)	67 (14)	66 (13)
Female gender, *n* (%)	200 (33.9)	202 (34.6)
BMI (kilograms divided by height in metres squared), mean (SD)	26.7 (6.6)	27.4 (7.1)
Chronic alcohol use, *n* (%)	115 (19.5)	126 (21.6)
Chronic psychotropic medication use, *n* (%)	177 (30.0)	166 (28.4)
Benzodiazepine and related medications	110 (18.6)	109 (18.7)
Neuroleptics	22 (3.7)	33 (5.7)
Antidepressants	72 (12.2)	70 (12.0)
Opioid medication	42 (7.1)	35 (6.0)
Tobacco use, *n* (%)	156 (26.5)	169 (28.9)
Liver cirrhosis with ascites or oesophageal varices, *n* (%)	34 (5.8)	37 (6.3)
Chronic renal replacement therapy, *n* (%)	9 (1.5)	10 (1.7)
Chronic respiratory insufficiency with home oxygen therapy, *n* (%)	42 (7.1)	51 (8.7)
NYHA class IV chronic heart failure, *n* (%)	16 (2.7)	23 (3.9)
Barthel score before admission, median (*Q*1–*Q*3)	100 (100–100)	100 (95–100)
Knauss chronic health status before admission, *n* (%)		
Normal health status	155 (26.3)	152 (26.0)
Moderate activity limitation	285 (48.3)	267 (45.7)
Severe activity limitation due to chronic disease	139 (23.6)	157 (26.9)
Bedridden patient	11 (1.9)	8 (1.4)
MacCabe class before admission, *n* (%)		
No fatal disease	363 (61.5)	348 (59.6)
Ultimately fatal disease	181 (30.7)	197 (33.7)
Rapidly fatal disease	46 (7.8)	39 (6.7)
At home without assistance before current hospital admission, *n* (%)	373 (63.2)	367 (62.8)
ICU admission SAPS II score (first 24 h), mean (SD)	54.4 (18.6)	53.6 (17.8)
ICU admission SOFA score (first 24 h), median (*Q*1–*Q*3)	9 (7–12)	9 (7–12)
Medical admission, *n* (%)	520 (88.1)	530 (90.8)
Norepinephrine at randomization, *n* (%)	312 (53.0)	318 (54.5)
Midazolam at randomization (*n*_1_ = 493, *n*_2_ = 496), *n* (%)	412 (83.6)	421 (84.9)
Propofol at randomization (*n*_1_ = 493, *n*_2_ = 496), *n* (%)	74 (15.0)	69 (13.9)
Severe sepsis, *n* (%)	50 (8.5)	57 (9.8)
Septic shock, *n* (%)	339 (57.5)	323 (55.3)
ARDS, *n* (%)	186 (31.5)	187 (32.1)
ICU primary diagnosis		
Pulmonary infection, *n* (%)	246 (41.7)	239 (40.9)
Abdominal infection, *n* (%)	49 (8.3)	54 (9.3)
Other Infection, *n* (%)	41 (6.9)	57 (9.8)
Cardiac failure or cardiogenic pulmonary oedema, *n* (%)	56 (9.5)	53 (9.1)
COPD exacerbation or acute asthma, *n* (%)	51 (8.6)	51 (8.7)
Acute pancreatitis, *n* (%)	9 (1.5)	13 (2.2)
Drug intoxication, *n* (%)	12 (2.0)	9 (1.5)
Metabolic disorder, *n* (%)	18 (3.1)	7 (1.2)
Trauma, *n* (%)	7 (1.2)	7 (1.2)
Acute stroke, *n* (%)	5 (0.8)	2 (0.3)
Miscellaneous, *n* (%)	96 (16.3)	92 (15.8)
Sedation level on the RASS scale at randomization^a^		
Very agitated, *n* (%)	1 (0.4)	3 (1.1)
Agitated, *n* (%)	4 (1.5)	1 (0.4)
Restless, *n* (%)	3 (1.1)	5 (1.9)
Alert and calm, *n* (%)	12 (4.5)	12 (4.6)
Drowsy, *n* (%)	12 (4.5)	12 (4.6)
Light sedation, *n* (%)	21 (7.9)	15 (5.7)
Moderate sedation, *n* (%)	30 (11.3)	23 (8.8)
Deep sedation, *n* (%)	63 (23.8)	66 (25.2)
Unarousable, *n* (%)	119 (44.9)	125 (47.7)

SD, standard deviation; BMI, body mass index; NYHA, New York Heart Association; *Q*1–*Q*3, 1st and 3rd quartiles; SAPS, Simplified Acute Physiology Score, SOFA, Sequential Organ Failure Assessment; ICU, intensive care unit, COPD, chronic obstructive pulmonary disease; ARDS, acute respiratory distress syndrome

^a^Sedation was measured using the RASS scale in 268 patients in the control group and 267 patients in the oversedation prevention group. The exact level on the RASS scale was not available for 3 patients in the control group and 5 patients in the oversedation prevention group.

### Outcomes

**Fig. 3:**
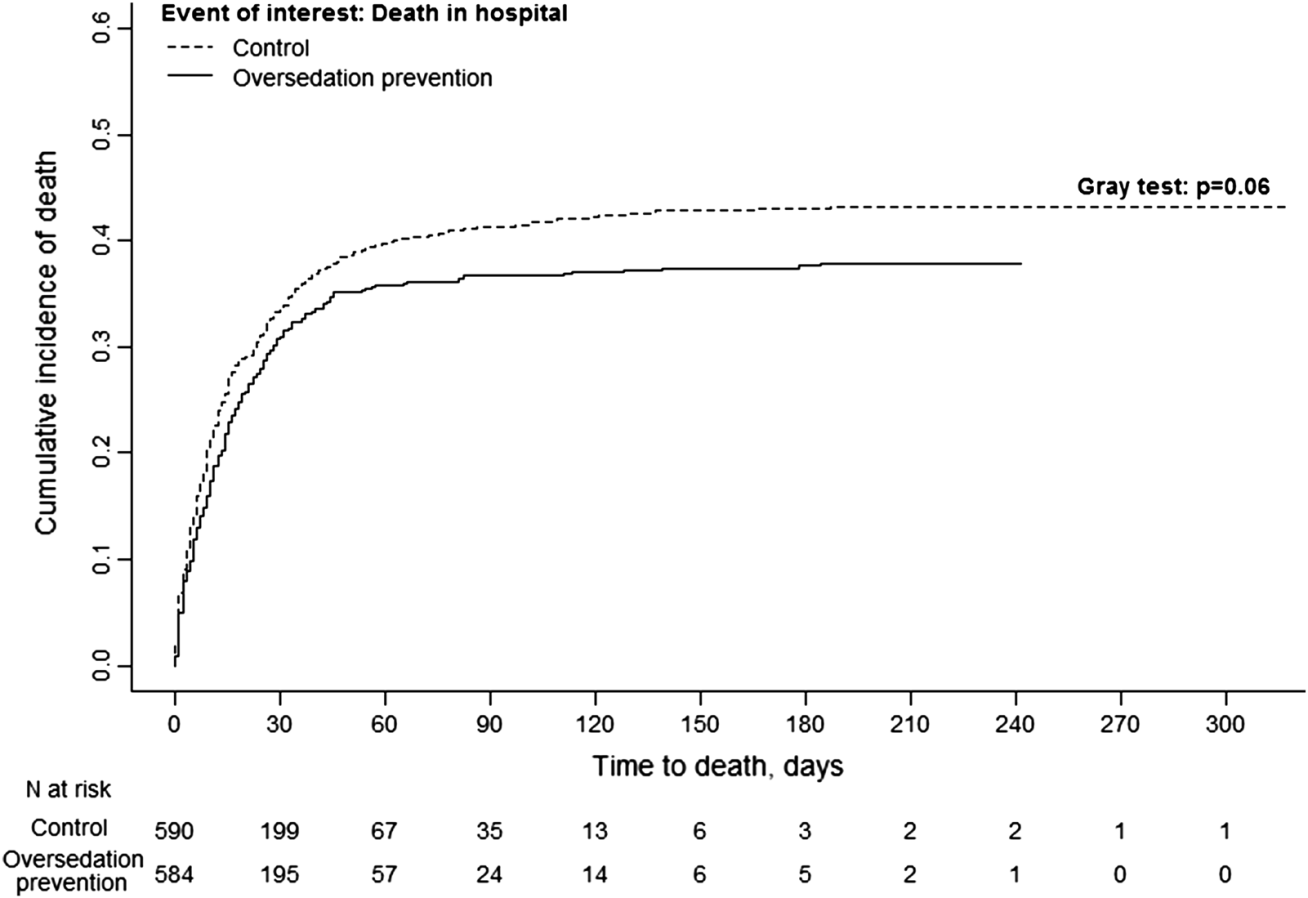
Cumulative incidence of deaths in the hospital. The cumulative incidence of hospital death did not differ significantly between the two groups (220 in oversedation prevention group vs. 253 in the control group): hazard ratio for the oversedation prevention group versus the control group, 0.85; 95% confidence interval, 0.71–1.01; *p* = 0.06. For the analysis of time from randomization to death in the hospital, alive hospital discharge was handled as a competing risk

At day-90, 230 patients (39.4%) had died in the OSP group and 261 (44.2%) in the control group, *p* = 0.09. Of note those mortality rates were far higher than the initial sample size calculation of the trial. There were also no significant differences in day-28 (Table 2), in-hospital (Fig. 3), and 1-year mortality between the two groups (Table 2). Cumulative dosages of intravenous propofol and midazolam were significantly lower in the OPS group (Table 2). Cumulative dosage of intravenous sufentanil was significantly lower in the OPS group, whereas there was no significant difference in cumulative dosages of other morphinics between the two groups (Table 2).

**Table 2:** Outcomes

	Control (*n* = 590)	Oversedation prevention (*n* = 584)	*P* value	Hazard ratio (95% confidence interval)
28-day mortality	198 (33.6)	177 (30.4)	0.24^a^	
90-day mortality	261 (44.2)	230 (39.4)	0.09^a^	
1-year mortality	296 (60.0)	267 (56.5)	0.26^a^	
Mechanical ventilation-free days at day 28 (days), median (*Q*1–*Q*3)	14 (0–24)	16 (0-24)	0.36^b^	
Ventilator-associated pneumonia, *n*	92	94	0.79^c^	1.04 (0.78; 1.38)
Mechanical ventilation ≥ 48 h, *n* (%)	425 (72.0)	418 (71.6)	0.86^a^	
Non-invasive ventilation after extubation, *n* (%)	152 (25.8)	177 (30.3)	0.08^a^	
Duration of non-invasive ventilation after extubation (days), median (Q1–Q3)	2 (1–4)	3 (2–4)	0.05^b^	
Tracheostomy, *n*	26	24	0.81^c^	0.93 (0.54; 1.62)
Delirium, *n*	232	230	0.99^c^	1.00 (0.84; 1.19)
Proximal weakness after awakening, *n*	193	208	0.26^c^	1.11 (0.92; 1.35)
Patients with intravenous midazolam, *n* (%)	464 (78.6)	419 (71.8)	0.01^a^	
Cumulative dosage of midazolam (mg), median (*Q*1–*Q*3)	263 (120–660)	218 (72–696)	0.03^b^	
Patients with intravenous propofol, *n* (%)	232 (39.3)	214 (36.6)	0.34^a^	
Cumulative dosage of propofol (mg), median (*Q*1–*Q*3)	2785 (645–7140)	1443 (120–4800)	< 0.001^b^	
Patients with intravenous morphinics, *n* (%)	501 (84.9)	482 (82.5)	0.31^a^	
Patients with IV sufentanil, *n* (%)	263 (44.6)	241 (41.3)	0.28^a^	
Cumulative dosage of sufentanil (µg), median (*Q*1–*Q*3)	930 (472–2592)	870 (280-2160)	0.04^b^	
Patients with IV fentanyl, *n* (%)	204 (34.6)	206 (35.3)	0.8^a^	
Cumulative dosage of fentanyl (µg), median (*Q*1–*Q*3)	4985 (2400–15,445)	4656 (1340–16,200)	0.29^b^	
Patients with IV morphine, *n* (%)	73 (12.4)	91 (15.6)	0.1^a^	
Cumulative dosage of morphine (mg), median (*Q*1–*Q*3)	17.5 (7–55)	20 (6–43)	0.69^b^	
Patients with IV remifentanil, *n* (%)	49 (8.3)	45 (7.7)	0.7^a^	
Cumulative dosage of remifentanil (µg), median (*Q*1–*Q*3)	14,400 (6000–28,800)	7200 (3000-19,200)	0.05^b^	
Self-extubation, *n*	48	70	0.03^c^	1.50 (1.04; 2.16)
Ventricular tachycardia or fibrillation, *n*	18	21	0.61^c^	1.18 (0.63; 2.11)
Acute coronary syndrome or myocardial infarction, *n*	7	8	0.77^c^	1.16 (0.42; 3.18)
Cardiac arrest, *n*	20	13	0.22^c^	0.65 (0.33; 1.31)

For comparison of time dependent events analyzed using competing risks models to take into account competing risks as death or extubation (e.g. ventilator associated pneumonia), no percentages are provided

For comparison of variables in post-randomization sub-group (e.g. ICU length of stay in survivors), no *P* values are provided

CI, confidence interval; Q1–Q3, 1st and 3rd quartiles; MV, mechanical ventilation; VAP, ventilator-associated pneumonia; NIV, non-invasive ventilation; ICU, intensive care unit; LOS, length of stay

^a^Variables compared using the chi-square test

^b^Variables compared using the Wilcoxon test

^c^Variables analyzed using competing risks models to take into account competing risks (as death, extubation, ICU discharge, …). For each of these outcomes, Gray test *P* value and hazard ratio (95% confidence interval) from competing risks models were presented

First spontaneous breathing trial occurred significantly earlier in the OSP group than in the control group (HR 1.18 [1.03–1.36], *p* = 0.015 (Additional file 1: Appendix 3, Figure). Similarly, successful extubation occurred significantly earlier in the OSP group than in the control group (HR 1.15 [1.02–1.31], *p* = 0.03 (Additional file 1: Appendix 3, Figure).

There was no significant difference in the other secondary outcomes, i.e. time to first sitting in a chair, time to first standing by the bed, presence of proximal muscle weakness, delirium, length of stay in the ICU (Table 2). Self-extubation was significantly more frequent in the OSP group than in the control group (70 vs. 48 events, HR 1.50 [1.04; 2.16], *p* = 0.03). Percentage of patients awake on a daily assessment between day 1 and day 7 are presented in the Additional file 1: Appendix 4.

## Discussion

In this multicentre randomized study, we were unable to show that a gradual multilevel bundle strategy to prevent oversedation could significantly reduce mortality of severely ill ICU patients requiring mechanical ventilation. There were no significant differences between the two groups in in-hospital and 1-year mortality. However, oversedation prevention resulted in significantly less use of intravenous midazolam and propofol, and significantly earlier weaning initiation and extubation. Last, the numerous limitations including early termination of the trial weaken the result interpretation.

We chose an OSP strategy centred on the identification of patients’ level of agitation, ventilator asynchrony, and pain, on a 4-level scale, with gradual on-demand responses, frequent reassessments, and promotion of alternatives to continuous around-the-clock intravenous hypnotics (midazolam or propofol) infusion. Interestingly, in the OSP algorithm, interventions were titrated only on patients’ needs to control pain, agitation, and ventilator asynchrony (except in the level 3), with no attempt to alter consciousness, even slightly, as a specific goal. Accordingly, the OSP strategy did not include the use of any sedation scale. Cumulative dosages of propofol and midazolam were significantly lower in the OPS group. We did not use dexmedetomidine as an alternative to continuous intravenous hypnotics because at the time of study design, the very recent commercialization of dexmedetomidine in France precluded homogeneous and optimal use among the participating centres [22–25].

Our study was unable to show that the OSP strategy reduced mortality compared to standard care in critically ill patients. Furthermore, mortality was high in the study population. More than 40% of the patients had died at 3 months and almost 60% at 1 year. These mortality rates were much higher than anticipated at study design and higher than those reported in previous studies on light sedation strategies [1–13]. This high mortality very likely reflects the severity of the acute conditions at ICU admission, as suggested by a SAPS II score higher than commonly reported in trials on sedation [5, 8, 10, 25, 26]. Old age and the low rate of post-operative admissions both could have contributed to the high SAPS II. Similarly, the percentage of patients on vasoactive drugs at randomization was high.

The severity of the acute conditions of our study population compared to previous studies suggests the inclusion of patients in real-life conditions. Demonstration of the positive impact of an intervention, such as a strategy to prevent oversedation, might be difficult in patients with particularly severe admission conditions and requires a larger sample of patients. In our study, less than half of the planned included patients were finally enrolled which undoubtedly makes the trial underpowered. Furthermore, the planned inclusion number was based on a mortality rate of 22% in the control group. There is such a high difference between the a priori postulated mortality rate in the control group, and the a posteriori observed one (which is much closer to 50%) that even in case we would have been able to recruit the planned 2720 patients, the real power of the trial would have been much lower than the 90% nominal power.

Despite the severity of the conditions of the study patients, and the above limitations, the OSP strategy resulted in significantly shorter mechanical ventilation duration. Similar findings have been observed in previous randomized studies on light sedation in less severely ill ICU populations. This finding is important, as physicians may be reluctant to adopt a light sedation strategy among the most severely ill patients. Indeed, as agitation and device removal may be perceived as particularly dangerous in this population, physicians may favour continuous intravenous sedation. The present trial did not show that oversedation prevention was associated with lower mortality, but it showed that it was associated with secondary benefits of faster weaning and extubation.

One explanation for shorter mechanical ventilation duration is provided in our study by a significantly shorter time to first spontaneous breathing trial. An adequate consciousness level is among the prerequisite criteria for physicians to initiate the weaning process leading to extubation, along with other criteria including absence of high-grade fever, low oxygen, positive expiratory end-pressure, and vasoactive drug needs [27]. A light sedation strategy might promote preservation of consciousness or early return to consciousness when other weaning criteria are met [28].

*Study limitations* The numerous limitations including the early termination and associated lack of power weaken the results.

We did not design a weaning protocol in the control group; patients were treated according to usual practice in the participating ICUs. Unfortunately, we do not have any data showing that the French ICU weaning guidelines were applied similarly in both groups. Physicians in the participating study centres might have unconsciously changed their practice over the study period, with a progressive implementation of some aspects of the OSP strategy in control patients, further reducing the difference in sedation practices between the two groups. Insufficient compliance with the relatively novel multilevel gradual intervention might also have reduced the difference in sedation practices between the two groups. A cluster randomization at the ICU level would have limited those group contamination issues; however, the risk of a selection bias associated with such a design was deemed greater and led us to choose an individual randomization scheme [29–32]. This point remains a strong limitation to interpret the secondary endpoints in this non-blinded study.

The gap between estimated 90-day mortality used for the sample size calculation and the higher mortality rates observed also represents an important limitation study further reducing the study power.

Another limitation is that we did not use a sedation scale to measure the effect of the OSP strategy on consciousness. This option was deliberately selected to avoid that clinicians would try to titrate IV sedatives to reach the common target of slightly altered consciousness in the intervention group, in which no specific alteration of consciousness should be targeted. Unfortunately, the surrogate markers for consciousness level used in the study (amounts of sedatives used, single daily assessment of consciousness base on a yes/no single-item scale, MV duration, etc.) all have their own limitations.

The oversedation prevention resulted in significantly less use of intravenous midazolam and propofol. Measuring doses of drug and showing a reduction in doses administered is not the same thing, but the increased rate of awake patients in the OSP group may imperfectly reflect the goal. Unfortunately, an explicit recording of implementation of multiple components of the OSP protocol could not be carried out in this large trial.

Of note, the lack of details regarding the previous alcohol consumption and the grouping within the large psychotropic category of medications with various mechanisms of action and side effects (benzodiazepines, neuroleptics, antidepressants, etc.) albeit pragmatic also represents a potential methodological limitation.

In summary, in this prospective randomized trial in severe critically ill patients requiring mechanical ventilation with early termination and under powering, we were unable to show that oversedation prevention significantly reduces mortality. However, it resulted in a significantly lower use of intravenous hypnotics, earlier time to spontaneous breathing trial, and reduced duration of mechanical ventilation. These last results should be interpreted with precaution regarding the several limitations of the trial including the early termination.

## Additional file

**Additional file 1.** Complementary outcome criteria, results and SRLF trial group contributors.

## Abbreviations

95% CI: 95% confidence interval
HR: hazard ratio
ICU: intensive care unit
OSP: oversedation prevention
