# Supplementary material for: Impact of oversedation prevention in ventilated critically ill patients: a randomized trial—the AWARE study
Source: Ann Intensive Care. 2018 Sep 21;8:93. doi: 10.1186/s13613-018-0425-3 (PMC6150862; doi:10.1186/s13613-018-0425-3)
Supplement: Supplementary file 1 — Additional file 1. Complementary outcome criteria, results and SRLF trial group contributors. [file 13613_2018_425_MOESM1_ESM.docx]

**Impact of oversedation prevention in ventilated critically ill patients: a randomized trial – The AWARE study**

**SRLF Trial Group**

**Additional file 1 : Complementary outcome criteria, results and SRLF trial group contributors**

**Appendix 1. Outcome criteria**

The primary study endpoint was mortality 90 days after randomization. Secondary endpoints were day-28, hospital and 1-year mortality, time from randomization to first spontaneous breathing trial, time to successful extubation (defined as absence of mechanical ventilation for 48 consecutive hours), mechanical ventilation-free days during the first 28 days after randomization, requirement for and duration of non-invasive ventilation after extubation, rate of ventilator-associated pneumonia, time to first sitting in a chair, time to first standing by the bed, presence of proximal weakness, time to first episode and number of days of delirium (assessed using the confusion assessment method for the intensive care unit (CAM-ICU)^(1)^), length of stay in ICU, number of days and amounts of IV hypnotics administered, and percentage of patients awake between Day 1 and Day 7 (see Appendix 4). The following safety criteria were also assessed: self-removal of drains or tubes (including the tracheal tube), ventricular tachycardia or fibrillation, acute coronary syndrome or myocardial infarction, and cardiac arrest.

Of note, delirium was assessed by physicians using the CAM-ICU daily during the first week, at day 14, at day 21 and at day 21.

**Appendix 2. Sedation scale used, and baseline sedation levels at randomization**

**Sedation scale used (Table 2-1)**

|  | **Control**  **(n=590)(a)** | **Oversedation**  **prevention**  **(n=584)** |
| --- | --- | --- |
| **RASS^(2)^, n (%)** | **268 (45.5)** | **267 (45.7)** |
| **Ramsay^(3)^, n (%)** | **119 (20.2)** | **108 (18.5)** |
| **Other, n (%)** | **82 (13.9)** | **68 (11.6)** |
| **None, n (%)** | **120 (20.4)** | **141 (24.1)** |

1. **Data about the sedation scale use (RASS, Ramsay, other or none) was unavailable for 1 patient in the Control group.**

**Sedation level on the Ramsayscale (3) at randomization (Table 2-2)**

|  | **Control**  **(n=119)** | **Oversedation**  **prevention**  **(n=108)** |
| --- | --- | --- |
| Patient anxious or agitated or both, n (%) | 3 (2.5) | 2 (1.8) |
| Patient cooperative, orientated and tranquil, n (%) | 4 (3.4) | 11 (10.2) |
| Patient responds to command only, n (%) | 14 (11.8) | 18 (16.7) |
| A brisk response to a light glabellar tap or loud auditory stimulus, n (%) | 34 (28.6) | 19 (17.6) |
| A sluggish response to a light glabellar tap or loud auditory stimulus, n (%) | 26 (21.8) | 20 (18.5) |
| No response, n (%) | 38 (31.9) | 38 (35.2) |

References

1. [Ely EW](https://www.ncbi.nlm.nih.gov/pubmed/?term=Ely%20EW%5BAuthor%5D&cauthor=true&cauthor_uid=15082703), [Shintani A](https://www.ncbi.nlm.nih.gov/pubmed/?term=Shintani%20A%5BAuthor%5D&cauthor=true&cauthor_uid=15082703), [Truman B](https://www.ncbi.nlm.nih.gov/pubmed/?term=Truman%20B%5BAuthor%5D&cauthor=true&cauthor_uid=15082703), [Speroff T](https://www.ncbi.nlm.nih.gov/pubmed/?term=Speroff%20T%5BAuthor%5D&cauthor=true&cauthor_uid=15082703), [Gordon SM](https://www.ncbi.nlm.nih.gov/pubmed/?term=Gordon%20SM%5BAuthor%5D&cauthor=true&cauthor_uid=15082703), [Harrell FE Jr](https://www.ncbi.nlm.nih.gov/pubmed/?term=Harrell%20FE%20Jr%5BAuthor%5D&cauthor=true&cauthor_uid=15082703), [Inouye SK](https://www.ncbi.nlm.nih.gov/pubmed/?term=Inouye%20SK%5BAuthor%5D&cauthor=true&cauthor_uid=15082703), [Bernard GR](https://www.ncbi.nlm.nih.gov/pubmed/?term=Bernard%20GR%5BAuthor%5D&cauthor=true&cauthor_uid=15082703), [Dittus RS](https://www.ncbi.nlm.nih.gov/pubmed/?term=Dittus%20RS%5BAuthor%5D&cauthor=true&cauthor_uid=15082703). Delirium as a predictor of mortality in mechanically ventilated patients in the intensive care unit. JAMA 2004, 29:1753-62.
2. Ely EW *et al.* Monitoring sedation status over time in ICU patients: reliability and validity of the Richmond Agitation-Sedation Scale (RASS). JAMA 2003, 289:2983-91.
3. Ramsay MA, Savege TM, Simpson BR, Goodwin R.. Controlled sedation with Alphaxalone-Alphadolone. BMJ 1974, 2:656-9

**Appendix 3.**

**
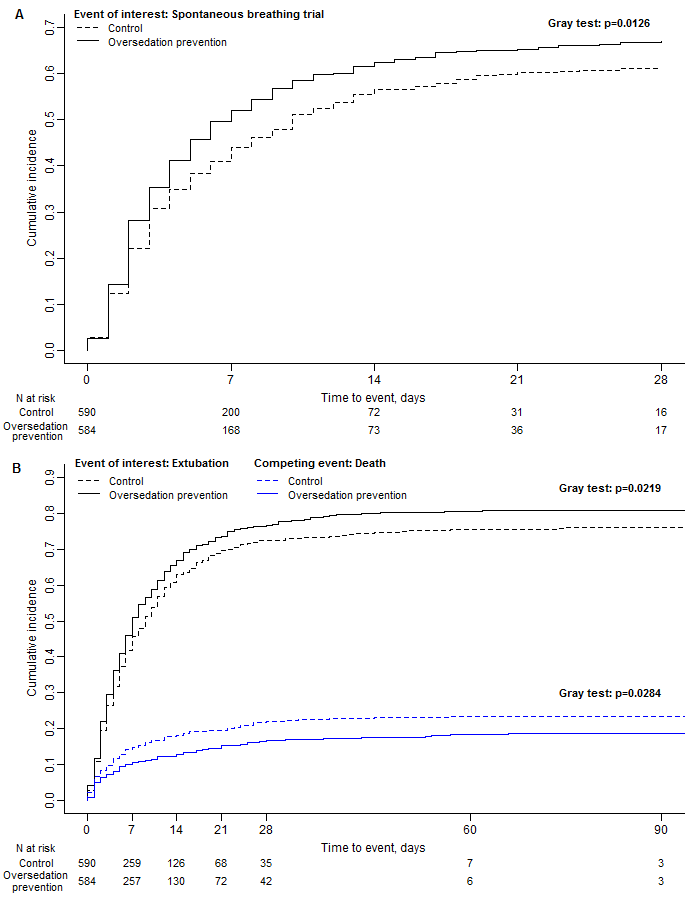
**

**Panel A. Time to first spontaneous breathing trial**

Curves represent cumulative incidence of spontaneous breathing trial success between randomization and day-28 in both groups (391 in the oversedation prevention group vs 361 in the control group, Gray test: p= 0.01). For the analysis of time from randomization to first spontaneous breathing trial, extubation and death were handled as competing risks. The hazard ratio from the competing risks model in the oversedation prevention group vs the control group was 1.18 (95% confidence interval 1.03 to 1.36).

**Panel B. Time to successful extubation**

Black curves represent cumulative incidence of successful extubation in both groups (447 in the oversedation prevention group vs 427 in the control group, Gray test: p= 0.02). For the analysis of time from randomization to successful extubation, death was handled as a competing risk (blue curves, 98 patients in the oversedation prevention group vs 130 in the control group, Gray test: p= 0.03). Curves were truncated at day-90. Three patients in each group experienced events after day-90 (until day-128); these patients were included in the analysis model.

**Appendix 4. Percentages of patients awake between Day 1 and Day 7**


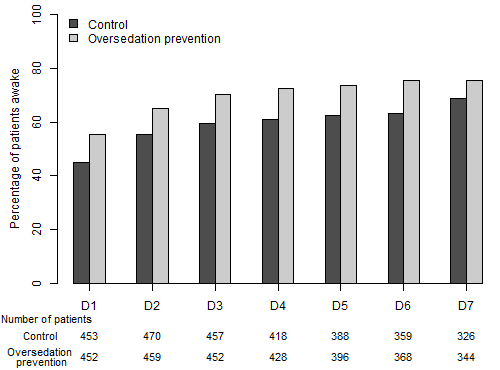


**Figure legend**

Percentage of patients with eyes open either spontaneously or to name and able to perform at least one of the following commands: "close your eyes", "look at me" or "nod your head" at a daily assessment in the morning

**Appendix 5. SRLF Trial Group contributors**

**Study coordination:** Bernard DE JONGHE

**Past and present members of the commission for epidemiology and clinical research, presidents and general secretary of the French intensive care society, writing committee:** Jérôme ABOAB; Nadia AISSAOUI (writing committee); Djillali ANNANE (past president); Corinne AUDOIN; Jean-Luc BAUDEL; Florence BROUARD; Alexandre CAMBONIE; Isabelle CAMILATTO; Karim CHERGUI; Vincent DAS; Daniel DA SILVA; Bernard DE JONGHE (writing committee), Nicolas DEVOS; Nicolas DEYE; Stephan EHRMANN (writing committee); Frédérique GANSTER; David GRIMALDI; Bruno GIRAUDEAU (writing committee); Emmanuelle GOURDIN; Antoine GROS; Olfa HAMZAOUI; Frédéric JACOBS; Antoine KIMMOUN; Jean-Claude LACHERADE; Bernard LAMBERMONT; Pierre-François LATERRE (past president); Julie LEGER (writing committee); Stéphane LEGRIEL; Lucas LIAUDET; Charles-Edouard LUYT (secretary); Alain MERCAT (president elect); Philippe MICHEL; Jean-Paul MIRA (president); Xavier MONNET (past secretary); Grégoire MULLER; Michael PIAGNERELLI; Gaëtan PLANTEFEVE; Jean REIGNIER (past president); Jean-Damien RICARD (past secretary); François VINCENT.

**Investigators:** ALIANE Jugurtha, CHU Gabriel Montpied, Réanimation Médicale Polyvalente, CLERMONT-FERRAND – PLOUVIER Fabienne, Hôpital Saint-Esprit, Réanimation Polyvalente, AGEN – MERCAT Alain, CHU d'Angers, Réanimation Médicale, ANGERS – MOHEBBI AMOLI Abolfazl, Hôpital Privé d'Antony, Réanimation Polyvalente, ANTONY – PLANTEFEVE Gaëtan, CH Victor Dupouy, Réanimation Polyvalente, ARGENTEUIL – PRAT Gwénaël, CHRU Cavale Blanche, Réanimation Médicale, BREST – FOUREL Didier, HIA Clermont Tonnerre, Fédération Anesthésie Réanimation Urgences, Réanimation polyvalente, BREST – CLEOPHAX Cédric, Hôpital René Dubos, Réanimation Médico-Chirurgicale, PONTOISE – CHERGUI Karim, CH Sud Francilien, Site Corbeil, Réanimation Polyvalente, CORBEIL-ESSONES – CARTEAUX Guillaume, CHU Henri Mondor, Réanimation Médicale, CRETEIL – ABOAB Jérôme, Hôpital Raymond Poincaré, Réanimation, GARCHES – REIGNIER Jean, CHD Les Oudairies, Réanimation Polyvalente, LA ROCHE SUR YON – TROCHE Gilles, CH de Versailles – Site André Mignot, Réanimation Médico-chirurgicale, LE CHESNAY – GUERIN Laurent, CHU Kremlin-Bicêtre, Réanimation Médicale, LE KREMLIN-BICETRE – GIRARDIE Patrick, Hôpital Roger Salengro, Réanimation Polyvalent, LILLE – VIVIER Emmanuel, Centre Hospitalier St Joseph St Luc, Réanimation Polyvalente, LYON – HERNU Romain, Groupe Hospitalier Edouard Herriot, Réanimation Médicale, LYON – OBBEE Philippe, Centre Hospitalier de Mâcon, Réanimation Polyvalente, MACON – DONETTI Laurence, GHIRM MONTFERMEIL, Réanimation, MONTFERMEIL – JACQUES Thierry, Polyclinique de Gentilly, Réanimation, NANCY – CRAVOISY-POPOVIC Aurélie, Hôpital Central, Réanimation Médicale, NANCY – BOULAIN Thierry, CHR d'Orléans Hôpital de la Source, Réanimation Médicale Polyvalente, ORLEANS – LU Qin, CHU La Pitié Salpêtrière, Réanimation polyvalente, PARIS – REUTER Danielle, Hôpital Saint Louis, Réanimation Médicale, PARIS – AZOULAY Elie, Hôpital Saint Louis, Réanimation Médicale, PARIS – CLAVIER Hervé, Institut Mutualiste Montsouris, Réanimation Polyvalente, PARIS – PICARD Walter, CH François Mitterand, Réanimation Polyvalente, PAU – DE JONGHE Bernard, CH de Poissy, Réanimation médico-chirurgicale, POISSY – ROBERT René, CHU de Poitier, Réanimation Médicale, POITIERS – CHOUQUER Renaud, CH de la Région d'Annecy, Réanimation Polyvalente, PRINGY – GIRAULT Christophe, CHU de Rouen; Hôpital Charles Nicolle, Réanimation Médicale, ROUEN – MARJOT-ZIMBACCA France, CH de St Brieuc, Réanimation Polyvalente, SAINT-BRIEUC – DA SILVA Daniel, Centre Hospitalier de St Denis Hôpital de la Fontaine, Réanimation Médico-Chirurgicale, SAINT-DENIS – MERAT Stéphane, HIA Bégin, Réanimation, SAINT-MANDE – QUENTIN Charlotte, CH de Saint-Malo, Réanimation Polyvalente, SAINT-MALO – HICTER Jean-François, Clinique Belledonne, Réanimation polyvalente, SAINT MARTIN D’HERES – SCHENCK Maleka, Hôpital de Hautepierre, Réanimation Médicale, STRASBOURG – DAURIAC Sandie, Hôpital Jean Bernard; CH de Valenciennes, Réanimation Polyvalente, VALENCIENNES – DESMARETZ Jean-Luc, CH Armentières, Réanimation, ARMENTIERES – HYVERNAT Hervé, Hôpital l’Archet, Réanimation Médicale, NICE – SOUMER Alexis, Hôpital Foch, Réanimation polyvalente, SURESNES – STOCLIN Annabelle, Institut Gustave Roussy, Réanimation Polyvalente – USCM, VILLEJUIF – RIGAUD Jean-Philippe, CH Diepp, Réanimation Polyvalente, DIEPPE – DUGUET Alexandre, CHU La Pitié Salpêtrière, Pneumologie et Service de Réanimation, PARIS – BODET-CONTENTIN Laetitia, CHRU de Tours; Hôpital Bretonneau, Médecine Intensive Réanimation, TOURS – AU Siu-Ming, Hôpital Ambroise Paré, Réanimation Médicale, BOULOGNE BILLANCOURT – ENA Sébastien, CH de Rodez; Hôpital Jacques Puel, Réanimation, RODEZ.
